# Balancing Selection on a Regulatory Region Exhibiting Ancient Variation That Predates Human–Neandertal Divergence

**DOI:** 10.1371/journal.pgen.1003404

**Published:** 2013-04-11

**Authors:** Omer Gokcumen, Qihui Zhu, Lubbertus C. F. Mulder, Rebecca C. Iskow, Christian Austermann, Christopher D. Scharer, Towfique Raj, Jeremy M. Boss, Shamil Sunyaev, Alkes Price, Barbara Stranger, Viviana Simon, Charles Lee

**Affiliations:** 1 Department of Pathology, Brigham and Women's Hospital, Boston, Massachusetts, United States of America; 2 Harvard Medical School, Boston, Massachusetts, United States of America; 3 Department of Microbiology, Mount Sinai School of Medicine, New York, New York, United States of America; 4 Department of Microbiology, Emory University, Atlanta, Georgia, United States of America; 5 Division of Genetics, Department of Medicine, Brigham and Women's Hospital, Boston, Massachusetts, United States of America; 6 Department of Epidemiology, Harvard School of Public Health, Boston, Massachusetts, United States of America; 7 Department of Biostatistics, Harvard School of Public Health, Boston, Massachusetts, United States of America; 8 Global Health and Emerging Pathogens Institute, New York, New York, United States of America; 9 Division of Infectious Diseases, Department of Medicine, New York, New York, United States of America; University of Washington, United States of America

## Abstract

Ancient population structure shaping contemporary genetic variation has been recently appreciated and has important implications regarding our understanding of the structure of modern human genomes. We identified a ∼36-kb DNA segment in the human genome that displays an ancient substructure. The variation at this locus exists primarily as two highly divergent haplogroups. One of these haplogroups (the NE1 haplogroup) aligns with the Neandertal haplotype and contains a 4.6-kb deletion polymorphism in perfect linkage disequilibrium with 12 single nucleotide polymorphisms (SNPs) across diverse populations. The other haplogroup, which does not contain the 4.6-kb deletion, aligns with the chimpanzee haplotype and is likely ancestral. Africans have higher overall pairwise differences with the Neandertal haplotype than Eurasians do for this NE1 locus (p<10^−15^). Moreover, the nucleotide diversity at this locus is higher in Eurasians than in Africans. These results mimic signatures of recent Neandertal admixture contributing to this locus. However, an in-depth assessment of the variation in this region across multiple populations reveals that African NE1 haplotypes, albeit rare, harbor more sequence variation than NE1 haplotypes found in Europeans, indicating an ancient African origin of this haplogroup and refuting recent Neandertal admixture. Population genetic analyses of the SNPs within each of these haplogroups, along with genome-wide comparisons revealed significant *F_ST_* (p = 0.00003) and positive Tajima's *D* (p = 0.00285) statistics, pointing to non-neutral evolution of this locus. The NE1 locus harbors no protein-coding genes, but contains transcribed sequences as well as sequences with putative regulatory function based on bioinformatic predictions and *in vitro* experiments. We postulate that the variation observed at this locus predates Human–Neandertal divergence and is evolving under balancing selection, especially among European populations.

## Author Summary

Natural selection shapes the genome in a non-random way, as an allele that contributes more to the reproductive fitness of a species increases in frequency within the population. Under balancing selection, a particular kind of natural selection, more than one allele increases in frequency in the population, likely due to a reproductive advantage of individuals carrying both alleles. Only a handful of loci have been well documented to evolve under balancing selection, with the *HBB* gene (sickle cell locus) being the best studied. Here, we report a non-coding (but putatively functional) locus that has maintained two divergent alleles in the human population since before the Human–Neandertal divergence and is therefore likely to be under balancing selection. These findings also provide a clear example for ancient African substructure.

## Introduction

Most functionally important genomic loci in modern humans, including the majority of exons are under negative (purifying) selection and consequently show little, if any, genetic variation. In contrast, other forms of selection, such as balancing or directional positive selection, occur less frequently. The identification of such selection entails the detection of genomic variants that show unexpectedly high population differentiation or deviation from the prevalent haplotype structure [1]–[6]. There are only a few loci in the human genome that have been shown to evolve under balancing selection [7]–[9]. Some of these genic regions include the HLA locus [10], *HBB* [11], *ERAP2* [12], *PTC* [13] and the *G6PD* [14] genes, as well as a number of regulatory regions [15]–[17]. One hallmark of balancing selection is that it maintains a high level of ancient variation over long periods of time [18], [19].

Two major concepts have arisen in the last decade regarding the substantial impact of ancient genomic variation in modern humans. The first is that Neandertals have contributed 1–4% of their genome to non-African populations [20] and Denisovans have contributed 4–6% of their genome to modern Melanesian populations [21], sometimes with adaptive consequences [22], [23]. The second concept is that by comparing entire genomes to one other, studies have shown the presence of ancient genetic substructure in Africa affecting numerous loci [24]. These two concepts shape our understanding of the evolutionary and demographic factors that maintain unusual patterns of variation at several loci among modern humans.

**Figure 1:**
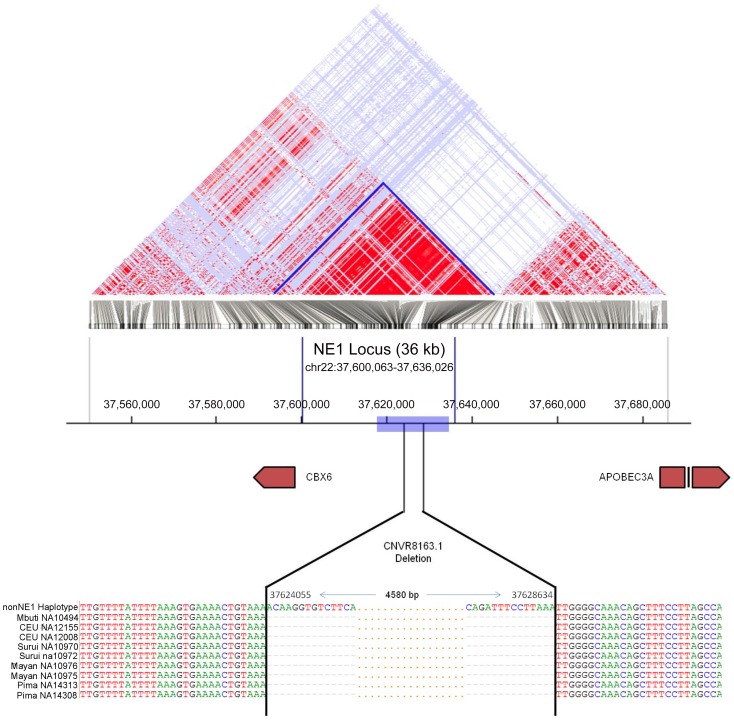
Map of the NE1 locus. The Linkage Disequilibrium (LD) block is determined using SNP data of the CEU population from the 1000 Genomes phase 1 data set using the HaploView program [55]. Red represents regions with a high degree of LD and a high log odds score (LOD; D' = 1, LOD>2). The blue box indicates the regions flanking the CNVR8163.1 deletion where statistics for genome-wide comparisons were calculated. The sequence alignments show the breakpoints of the CNVR8163.1 deletion as determined by PCR amplification followed by Sanger sequencing. The first sequence represents human reference (NCBI36/hg18), and the following 9 DNA sequences are from individuals with the CNVR8163.1 deletion. Coordinates 37,624,055 and 37,628,634 mark the breakpoints of this deletion on chromosome 22. “-” in the alignment identifies missing nucleotides in these individuals, and “.” depicts nucleotides which were not shown in that interval for illustrative clarity purposes.

Here, we present a locus, *NEandertal 1* (NE1), that encompasses a common copy number variant (CNV) [25]–[29], which appears to also be present in both Neandertal and Denisovan genomes and shows signatures of non-neutral evolution. The CNV exists as a 4.6 kb deletion polymorphism approximately 50 kb upstream of the *APOBEC3* locus, is common among Eurasians and resides in a well-defined 36 kb haplotype block (Figure 1). We have investigated the demographic and evolutionary forces that shape the variation at this locus and postulate that this locus harbors functional variation that predates the Human-Neandertal ancestor and has evolved under non-neutral, potentially balancing, selection.

## Results

### Characterization of a distinct haplogroup

**Figure 2:**
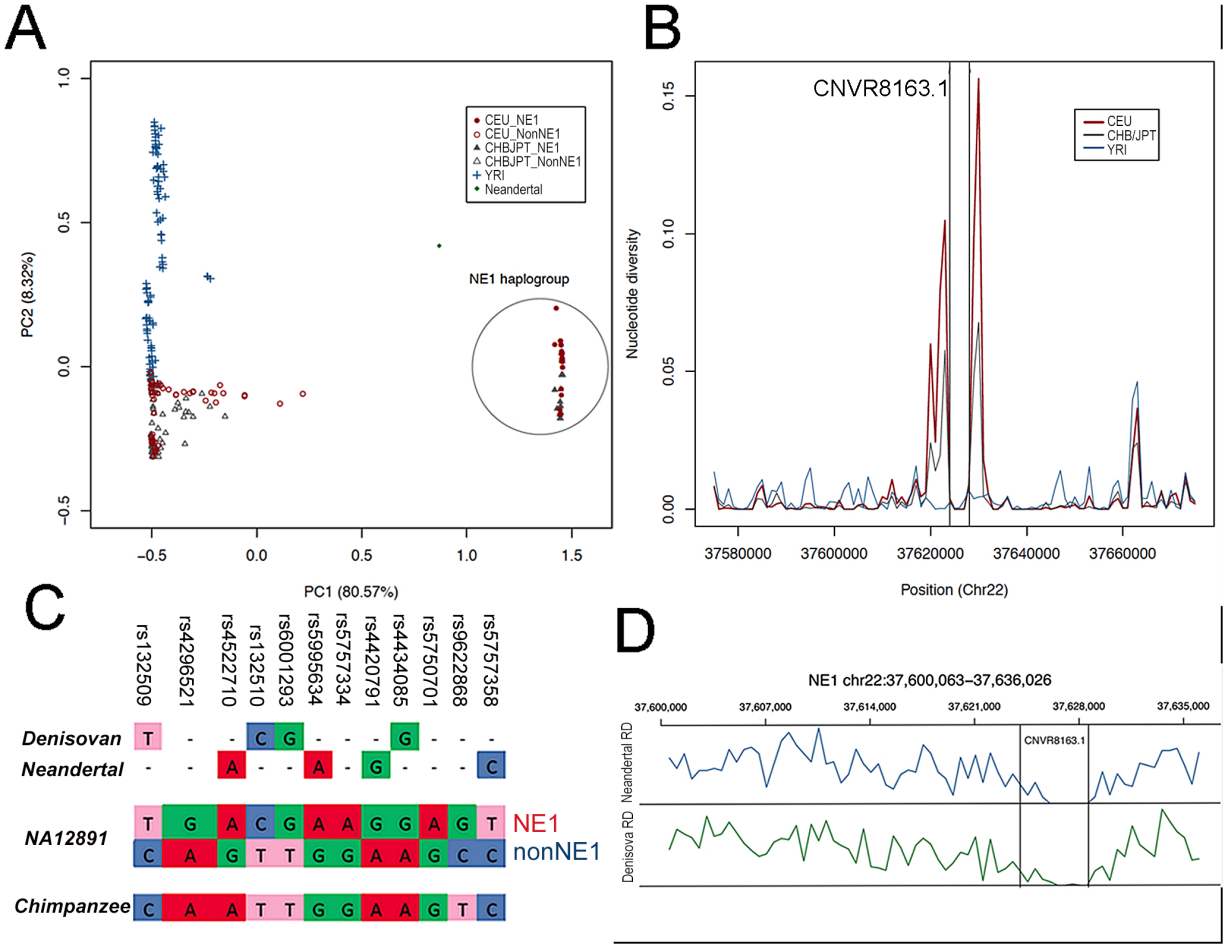
NE1 haplotypes share ancestry with Neandertals. (A) The principal component analysis (PCA) of the SNP haplotypes from CEU, CHB/JPT and YRI populations indicates population substructure between African and Eurasian populations, as well as within Eurasians. Please note the separation of NE1 and NonNE1 haplogroup. (B) Nucleotide diversity (π) within populations is depicted. For most of the genomic segments, π is higher within the YRI population (*blue line*) when compared to Eurasian populations (*red line*). However, there is an increase of π for Eurasian populations at the NE1 locus, surrounding the CNVR 8163.1 deletion. (C) The evaluation of 12 SNPs (that separate NE1 and nonNE1 haplogroups) in the CEU sample, NA12891, as well as Denisovan, Neandertal and chimpanzee consensus haplotypes. (D) Normalized read-depth of the Neandertal and Denisovan sequences across the NE1 locus. Please note the drop of the sequence read-depth to 0 for the region corresponding to the human CNVR8163.1 deletion.

To understand the genomic composition upstream of the *APOBEC3* locus, we first examined the phase I SNP data from the 1000 Genomes Project [30] and identified an unusually strong linkage disequilibrium (LD) block spanning approximately 36 kb (NE1 locus, hg18 - chr22:37,600,063–37,636,026) (Figure 1). This LD block is evident in Eurasian (CEU and CHB/JPT) populations but is absent in the Yoruban (YRI) population (Figure S1). Even though long stretches of LD can be indicative of selection, high LD can also result from a lack of recombination in the absence of selection [31], [32]. We conducted a principal component analysis (PCA) and found two distinct haplogroups (Figure 2A). We further identified 12 SNPs that can be used to distinguish these two haplogroups. Using Conrad et al. [33] and HapMap 3 [34] CNV genotypes, we identified a deletion polymorphism (CNVR8163.1) that is in perfect LD with these 12 defining SNPs so that one haplotype cluster contains the deletion and the other does not (Table S1). We sequenced across the putative breakpoints of this deletion in eight individuals and mapped the breakpoints to a 4,580 base pairs (bp) segment (hg18 – chr22: 37,624,055–37,628,634; Figure 1). This deletion polymorphism, along with the 12 defining SNPs, defined a distinct haplogroup, which we termed NE1. The nonNE1 haplogroup harbors the intact 4,580 bp segment. Using the phase 1 data from the 1000 Genomes Project (www.1000genomes.org), we identified 266 additional samples that harbor at least one chromosome with the deletion and the SNPs characteristic for the NE1 haplogroup (Table S2).

### Ancient African origins of the NE1 haplogroup

To investigate the overall amount of genomic variation at the NE1 locus, we plotted the average nucleotide diversity (π) [35] for 1000 bp bins across this locus, as well as for its flanking regions (+/−20 kb) (Figure 2B). π is a measure of the level of pairwise nucleotide differences between haplotypes within a population and can be used to compare variation in a population at a particular locus. For the majority of genomic loci, π is higher among YRI than among CEU (European ancestry) and CHB/JPT (Chinese/Japanese ancestry) populations [30]. However, there is a marked increase in π among Eurasians, but not in YRI, for the NE1 locus especially around the regions flanking CNVR8163.1 (Figure 2B). To test the statistical significance of this observation at a genome-wide level, we calculated π for 286,685 windows (10 kb) across the entire human genome and compared it with the π observed in two 5 kb regions flanking CNVR8163.1. We observed that both π and the number of segregating sites at the NE1 locus are significantly higher than expected by chance as shown by genome-wide simulation studies (p = 0.00050, Figure S2).

Such unusual nucleotide diversity has previously been attributed to admixture from archaic hominins, as they specifically affect non-African populations [20], [21]. We therefore examined whether the NE1 haplogroup clustered with the orthologous sequence in the Neandertal reference genome. Of the 12 SNPs that can be used to distinguish the NE1 and nonNE1 haplogroups, the SNPs that define the NE1 haplogroup aligned well with both the Neandertal and Denisovan orthologous sequences, whereas the chimpanzee consensus haplotype contain SNPs that are more similar to the nonNE1 haplogroup sequence (Figure 2C). Extending this analysis to 209 SNPs within the NE1 locus, we found that the Neandertal haplotype is more similar to CEU haplotypes than to YRI haplotypes (Mann-Whitney test, p<2.2e-16, Figure S3). Finally, read-depth analyses of the Neandertal and Denisovan sequences across the CNVR8163.1 deletion interval supports the notion that this sequence is homozygously deleted in sequenced ancient hominins, but not in the chimpanzee reference sequence (Figure 2D). Since the sample size for available archaic hominin genomes is extremely small, we cannot rule out the possibility that some Neandertals (and Denisovans) may carry the nonNE1 haplotype.

**Figure 3:**
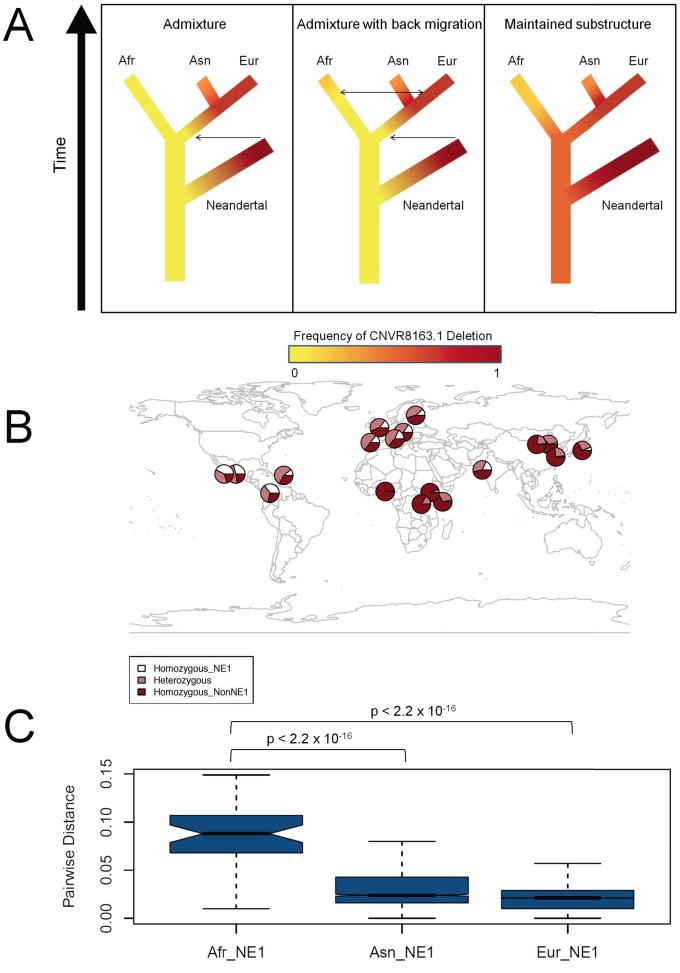
Ancient African origins of the NE1 haplogroup. (A) Models of scenarios that could lead to NE1 haplotypes observed in humans and Neandertals. The frequency of the NE1 haplogroup is depicted in red and the frequency of the nonNE1 haplogroup in yellow. The red corresponds to higher frequencies, whereas yellow corresponds to lower frequencies of the NE1 haplotypes in the population. The arrows represent the direction of possible admixture events. The left panel represents a model, under which the NE1 haplotypes admixed into Eurasian populations (Asn and Eur) after Human-Neandertal divergence. The second model, which is depicted in the central panel, is similar to the first model, except with the addition of more recent back migration of Eurasian NE1 haplotypes into Africa (Afr). The right panel shows the third model, under which the NE1 haplotypes among humans are explained by persistence of ancient African substructure. All these scenarios were based on the assumption that the NE1 haplotype occurs at high frequency or is fixed in the Neandertal population given that the available Neandertal sequences align well to the NE1 haplotype. (B) Geographical distribution of the NE1 haplogroup. We estimated the proportion of chromosomes that carry the CNVR8163.1 deletion from various sources described in *Materials and Methods*. The dark red portion of each circle represents the frequency of the homozygous nonNE1 genotypes, the white represents the homozygous NE1 genotypes and the light red represents the frequency of heterozygote genotypes. Note the existence of the NE1 haplotypes (i.e., as heterozygotes, *light* red) among sub-Saharan African populations (e.g., LWK and ASW) as well as the high frequency of heterozygotes (*light red*) in the European populations. (C) The pairwise distances between the African (Afr) NE1 haplotypes, the Asian (Asn) NE1 haplotypes, and the European (Eur) NE1 haplotypes, calculated using phase 1 data from the 1000 Genomes Project. p-values were calculated by the Mann-Whitney test.

Several scenarios can be envisioned to explain the unusual genetic variation observed at the NE1 locus: (1) recent Neandertal admixture exclusively with Eurasian populations, (2) back migration to Africa from Eurasia after Neandertal admixture with Eurasian populations, and (3) ancient African substructure maintained since before Human-Neandertal divergence (Figure 3A). We determined the frequency of the NE1 haplotypes among four African populations (YRI, ASW [African ancestry in Southwest USA], MKK [Maasai in Kinyawa, Kenya] and LWK [Luhya in Webuye, Kenya]) from the HapMap 3 dataset [34] and the 1000 Genomes Project [30] to distinguish between these three scenarios (Figure 3B). For this, we utilized the deletion genotypes of CNVR8163.1, which define the NE1 haplogroup. To ensure accuracy, we verified that HapMap 3 genotypes of this CNV were 99.5% concordant for individuals also genotyped by Conrad et al. [33]. Our results revealed moderate allele frequencies of CNVR8163.1 in some of the sub-Saharan African populations (0.27% in YRI, 8.19% in MKK, 2.78% in LWK and 18.04% in ASW, Table S2). To verify the presence of NE1 haplotypes in other sub-Saharan African populations, we used the phased haplotype data from the Human Genome Diversity Project (HGDP) [36]. In this dataset, six SNPs within the NE1 locus (rs11913682, rs4361209, rs132500, rs2142836, rs469987, rs2413552) were used to successfully categorize the haplotypes in 1190/1192 individuals into NE1 or nonNE1 haplotypes (Figure S4). We found, moreover, that 4 out of 30 (13%) of the Mbuti pygmy haplotypes belonged to the NE1 haplogroup and we obtained sequence confirmation of the CNVR8163.1 deletion in a Mbuti pygmy sample, NA10494 (Figure 1).

The presence of African NE1 haplotypes does not support the first scenario of exclusive Neandertal admixture with Eurasian populations. Recent reports have suggested that Neandertals and Denisovans contributed their genetic material to present-day Eurasian populations and Melanesians, respectively [20], [21]. However, the variation that we observe at the NE1 locus is not consistent with direct archaic hominin admixture as discussed in these publications. We did not consider Neandertal admixture into ancient African populations because of paleoanthropological studies that only report interactions between Neandertals and modern humans outside of Africa [37].

The second scenario assumes back migration into Africa from Eurasian populations after the admixture of Neandertal with Eurasian populations [38]. If such admixture occurred, the African NE1 haplotypes should represent a subset of Eurasian NE1 haplotypes. To test this, we again analyzed the phase 1 data of the 1000 Genomes Project, which includes 338 haplotypes from three African populations. Using this dataset, we found that variation within African NE1 haplotypes is significantly higher than variation within Asian and European NE1 haplotypes (p<10^−15^, Figure 3C, Figure S5). This result indicates that African NE1 haplotypes have a longer coalescence and, as such, the presence of the NE1 haplogroup among modern Africans cannot be explained by simple back migration and admixture of Eurasian haplotypes to African populations. Furthermore, the Mbuti pygmys are an extremely isolated population and yet we observed the CNVR8163.1 deletion (hence, NE1 haplotype) within this population. We have also observed the deletion in the available Denisovan genome, which further complicates the admixture followed by back-migration scenario, as this hominin species is thought to have only contributed genetic material to South East Asian populations. Although unusual migration and bottleneck scenarios can not be completely excluded, our data is not consistent with genetic variation at this locus being a result of back migration into Africa from Eurasian populations after the admixture of Neandertal with Eurasian populations.

The third scenario represents the persistence of an old African substructure at the NE1 locus before the Human-Neandertal divergence (Figure 3A). This scenario explains the presence of NE1 haplotypes (that are similar to the Neandertal haplotype) among modern human populations as well as the deep, distinct lineages observed among African NE1 haplotypes. To corroborate this conclusion, we estimated the coalescence of NE1 haplotypes through network analysis (Figure S6) and found a coalescence time of between ∼437 K and ∼993 K years before present (YBP) for African NE1 haplotypes and ∼134 K YBP and ∼304 K YBP for European NE1 haplotypes. These observations collectively suggest that the most parsimonious explanation for the observed variation at the NE1 locus is that the NE1/nonNE1 haplogroups arose after the human-chimpanzee common ancestor, but before the Human-Neandertal split in Africa. As such, the variation at the NE1 locus has persisted within ancient African substructure and later spread to non-African populations.

### The NE1 locus has likely evolved under balancing selection

**Figure 4:**
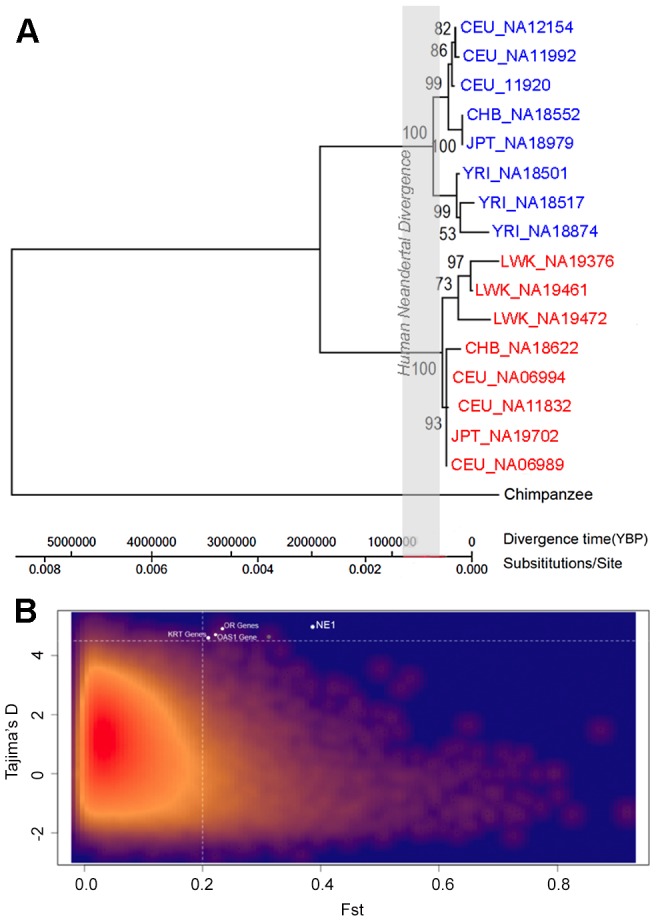
Selection acting on the NE1 locus. (A) Maximum likelihood tree based on select NE1 *(red)* and nonNE1 *(blue)* haplotypes, with the chimpanzee haplotype as an outgroup. The gray-box indicates the estimated interval for the Human-Neandertal divergence between 400,000–800,000 years ago [51]. Note that the coalescence at this locus is extremely long and very unlikely to have evolved under neutral conditions as modeled here. (B) Comparison of *F_ST_* and Tajima's *D* values of 10 kb intervals across the human genome. The red to dark blue gradient indicates decreased density of observed events at a given location in the graph. The NE1 locus, and other loci with similar profiles, are highlighted in white.

Since we ruled out admixture with archaic humans as an explanation for the unusual genetic variation observed for the NE1 locus, we hypothesized that selection may be acting on this genomic region. Indeed, the extreme divergence between haplogroups and the unusual nucleotide variation are consistent with the notion of non-neutral evolution, specifically, balancing selection, acting on the locus (Figure S6). To further scrutinize the nature of selective forces acting on the NE1 locus, we used the Tajima's *D* test, to assess for potential deviation from neutrality [39]. For this purpose, we focused on the regions flanking the CNVR8163.1 deletion in order to be consistent with our above-described analysis of π. Specifically, positive values of Tajima's *D* test indicate an excess of common variants compared to the neutral expectation within a population and is interpreted as one of the signatures of balancing selection. We observed significantly positive values for the Tajima's *D* statistics at the NE1 locus for CEU (3.54, p<0.01), FIN (Finnish individuals from Finland, 3.61, p<0.01), GBR (British individuals from England and Scotland, 3.415, p<0.01) and TSI (Tuscan individuals from Italy, 3.59, p<0.01) (Table S3). It is important to note that even though population size reductions can create positive Tajima's *D* values, these European populations have actually been subject to recent rapid population expansion [40]–[42], making it unlikely that the positive values of *D* observed at the NE1 locus are due to demographic events. To further support these observations, we measured Tajima's *D* across the entire genome for the CEU population, using 10 kb windows. We found that Tajima's *D* around the CNVR8163.1 deletion is a clear genome-wide outlier (p = 0.00003, Figure 4B, Figure S7). To further investigate the evolutionary history of this locus, we quantified population differentiation, *F_ST_*, which is a ratio of the genetic variation *among* populations to the genetic variation *within* populations. *F_ST_* values for the NE1 locus are generally elevated for most of the inter-continental comparisons (Table S4). A genome-wide comparison of *F_ST_* between CEU and YRI identifies the NE1 locus as a significant outlier (p = 0.00285, Figure S8). Taken together, Tajima's *D* and *F_ST_* analyses provide evidence that the two distinct haplogroups at the NE1 locus have evolved under non-neutral conditions.

High linkage disequilibrium (LD), due to lack of recombination, may affect the values of π, Tajima's *D* and *F_ST_* values and as such, they provide interdependent signatures of selection. Indeed, when we compared average pairwise LD between SNPs (*R* ^2^) in 10 kb windows across the genome, we found that LD weakly, but significantly, correlates with π (p<0.001, Pearson correlation coefficient (PCC) = 0.478) and Tajima's *D* (p<0.001, PCC = 0.455), but not with *F_ST_* (PCC = 0.052). To further establish the evolutionary forces acting on the NE1 locus, we repeated our genome-wide comparison for the loci within the 10 kb windows that show high LD (99^th^ percentile, *R* ^2^>0.59), as well as those that have a high number of segregating sites (99^th^ percentile, >263). The results confirmed our previous observations that the NE1 locus show significantly higher Tajima's *D*, even when compared to other genomic regions that have high LD (p = 0.0035) and a high number of segregating sites (p = 0.0011).

We also conducted a Hudson-Kreitman-Aguade (HKA) test [43] to determine whether the increased nucleotide diversity at the NE1 locus is due to balancing selection. This test compares within-species diversity to between-species divergence and has been used to test for balancing selection [e.g., 12]. The test assumes that under neutral evolution, the within-species polymorphism for at least two different loci is comparable to each other once normalized for respective between-species divergences observed at each locus. A locus under balancing selection would show higher than expected within-species variation as compared to neutrally evolving loci. We carried out a maximum likelihood HKA test by comparing the NE1 locus and 99 neutrally evolved loci randomly chosen at the whole genome level, using chimpanzee as the outgroup (Table S5). Our results show that there are more than expected segregating sites at the NE1 locus within the CEU population (p<0.01), further supporting the notion that the variation at this locus has evolved under balancing selection.

Furthermore, we performed a genome-wide investigation to identify regions that show π (>0.002), LD (*R* ^2^>0.5), Tajima's *D* (>4.5) and *F_ST_* (>0.2) similar to that of the NE1 locus (Figure 4B). We identified four other regions in the entire human genome that have a pattern similar to that of the NE1 locus (Table S6). Interestingly, three of these regions either overlap or are adjacent to environment interaction genes, such as the olfactory receptors, the innate immunity gene, *OAS1*, or the keratin associated proteins involved in hair formation. Indeed, a recent study reported that *OAS1* shows signatures of both Neandertal and Denisovan admixture [44], suggesting that loci that cluster with NE1 may have unusual evolutionary histories.

### Functional analysis of the genomic variation at the NE1 locus

**Figure 5:**
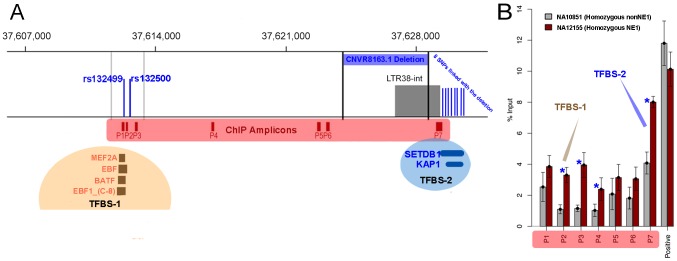
The regulatory functions of NE1 locus. (A) The LTR region was determined using the Repeat Masker Track version 3.2.7 [63]. Dark red boxes indicate the location of amplicons for ChIP qPCR. TFBS-1 and TFBS-2 refers to the two predicted transcription factor binding sites that were predicted by the latest ENCODE project data release, available in UCSC Genome Browser for the hg19 assembly. The CNVR8163.1 deletion polymorphism is flanked by black vertical lines. The blue vertical lines indicate the approximate locations of SNPs that differentiate between NE1 and nonNE1 haplogroups and overlap the TFBS-1 and TFBS-2 transcription factor binding sites. The 8 SNPs that overlap with TFBS-2 are from 5′ to 3′, rs132525, rs4306795, rs4434085, rs5750701, rs35853418, rs6001308, rs6001309, and rs9622868) (B) Chromatin immunoprecipitation quantitative PCR (ChIP-qPCR) results across the NE1 locus for representative samples NA12155 and NA10851 that belong to NE1 and nonNE1 haplogroups, respectively. The locations of the amplified segments (P1–P6) are shown in dark red rectangles in (A). The positive control primers amplify a segment within *BCL6* gene on chromosome 3 that is known to have high H3K4me2 occupancy. The blue stars indicate significant differences in qPCR amplification between NE1 and nonNE1 haplotypes (p<0.01). The brown and blue arrows indicate qPCR primers that are closest to the predicted transcription binding sites (P1, P2, P3 for TFBS-1 and P7 for TFBS-2). Overall, our results demonstrate that H3K4me2 is enriched in NA12155 cells, which harbor the NE1 deletion as compared to NA10851 cells which do not have the deletion (data plotted represents the average of four replicate experiments ± Std. Error).

We hypothesize that the two NE1 haplogroups have been maintained under balancing selection because of their putative regulatory function. To investigate this possibility, we looked for predicted regulatory elements within the locus, using data produced by the ENCODE project (Transcription Factor ChIP-seq tracks, [45]). In this dataset, we found two regions within the NE1 locus that bound to several transcription factors. We named these regions transcription factor binding sites 1 and 2 (TFBS-1 and TFBS-2, see also Figure 5A). Interestingly, there are a total of 10 SNPs that differentiate between the NE1 and nonNE1 haplogroups and reside within TFBS-1 or TFBS-2 (Figure 5A).

We conducted chromatin immunoprecipitation (ChIP) assays, followed by quantitative PCR (qPCR), for several positions across the NE1 locus to assess for histone H3 lysine 4 dimethylation (H3K4me2) enrichment. H3K4me2 is enriched in *cis* regulatory regions and was recently suggested to play a role in activating tissue specific gene expression [46]. Our results show that there is high H3K4me2 occupancy across the locus and that the occupancy remains consistently higher for NA12155 (homozygous NE1) as compared to NA10851 (homozygous nonNE1) (Figure 5B). Furthermore, we observed a significant difference between the H3K4me2 occupancy between NE1 and nonNE1 haplotypes at and around both transcription factor binding site regions (p<0.01, Figure 5B).

The 4.6 kb deletion in the NE1 haplotype removes a section of an endogenous retrovirus (ERV) element. Using a pGL3 vector-based luciferase reporter assay in HEK 293T cells, we found a short segment downstream from the nonNE1 haplotype (“Deleted LTR nonNE1”) that has promoter activity compared to the corresponding segment obtained from the NE1 haplotype (“Deleted LTR NE1”; p<0.001, Figure S9). However, further inquiry is warranted to fully understand the regulatory impact of this segment.

To identify potential gene targets of the putative regulatory sites within the NE1 locus, we performed a genome-wide *cis*- and *trans*- expression quantitative trait loci (eQTL) analysis in the three populations (CEU, CHB/JPT, YRI) using data from another study [47]. While, we observed several putative associations of SNPs at the NE1 locus affecting the expression of genes, such as *MGAT3*, *ATF*, *APOBEC3F* and *PLA2G6* (nominal p<0.001, Figure S10), no SNP-gene associations were considered significant after conservative multiple hypothesis testing.

## Discussion

Non-coding regulatory variation may be a major contributor to phenotypic variation [28] and are thought to be under strong selection among humans [48]. Only a handful of loci have been clearly shown to evolve under balancing selection [15]–[17]. In this study, we have identified a copy number variant, and its surrounding haplotype block, which shows highly atypical genetic structure within and among human populations and is likely under balancing selection.

There are two transcription factor binding site regions within the NE1 locus: TFBS-1 is upstream of the deletion polymorphism while TFBS-2, which is a target of SETDB1 and KAP1, is less than 1 kb downstream of the CNVR8163.1 deletion. KAP1 (also known as TRIM28) is a well-known transcriptional repressor that mediates its activity by recruiting a complex that also includes histone methyltransferase SETDB1 [49]. Of note is that KAP1 mediates silencing of both exogenous and endogenous retroviruses in embryonic stem cells [50]. Given that there are no known genes within the NE1 locus, it is unlikely that either region acts as a promoter. Instead, we speculate that these transcription factor binding sites may regulate transcription through long distance interactions. It is important to note that several of the SNPs that set apart the NE1 from nonNE1 haplotypes also change the sequence context of the transcription factor binding sites mentioned above. These SNP changes could explain the differential activity of active histone binding as measured by ChIP-qPCR. As such, it is attractive to speculate that these differences in regulatory activity may be the main target of the adaptive pressures acting on this locus but further functional characterization is required.

In cases of balancing selection, one usually finds an adaptive advantage of heterozygotes. Indeed, a considerable number of European populations show very high frequency of heterozygotes (>40%) and some populations, including Tuscans (TIS), Mexicans (MEX) and Puerto Ricans (PUR) show higher than 45% frequency of heterozygotes (Figure 3B). Moreover, the high *F*_ST_ values observed at this locus suggest that the strength of this force varies between different geographical regions.

Recent studies showed the existence of variation among modern humans that has persisted through ancient substructure [24]. Such substructure may account for some of the signals of the recently identified Eurasian hominin introgression [51]. The unusual nucleotide variation at the NE1 locus resembles signatures of Neandertal admixture to the modern Eurasian gene pool [e.g., 52]. If this variation were not detected among African populations, an argument would have been made for ancient hominin admixture to explain the observed variation. However, based on its presence in African population as well as previous theoretical insights [18], [19], we surmise that the NE1 and nonNE1 haplotypes were maintained by long-term balancing selection and most likely originated before the Human-Neandertal divergence. Future genome-wide scans for balancing selection, in genomic segments that were previously explained by admixture from archaic hominins, are warranted. The results of such studies will likely increase the number of known regions where balancing selection is acting and identify ancient variation that was previously attributed to archaic hominin admixture.

## Materials and Methods

### Quantitative analyses

The genotype data that we used for the majority of our quantitative analyses were from the data release 20100804 of the 1000 Genomes Project Phase 1 (http://www.1000genomes.org/data). The phased genotypes were processed from VCF (Variant Call Format) files by VCFtools [30], where the phased haplotypes were determined using the *IMPUTE2* software [53]. We further performed haplotype phasing inference and genotype imputation by *BEAGLE 3.0* [54] with default parameter settings. The common phased haplotypes from *IMPUTE* and *BEAGLE* that did not overlap with the CNVR8163.1 deletion were used for further analysis. The linkage disequilibrium (LD) analysis for the NE1 locus and its neighbor region, spanning ∼145 kb was carried out with Haploview 4.1 [55]. The LD block was determined to be ∼36 kb spanning a region between SNPs rs115660277 to rs5757362, using a stringent LD threshold. The nucleotide diversity (π) [35] in this region was estimated on a 1 kb sliding window size. Principal components analysis (PCA), implemented in the *R* package (http://www.r-project.org/), was applied to identify structure in the distribution of genetic variation across multiple geographical locations and ancestral backgrounds. The network analysis were conducted by Network 4.610 [56] and the coalescent to ancestral nodes on the network was calculated by the same software as described in [57].

### Population genetic analyses

To estimate worldwide geographical distribution of CNVR8163.1 deletion genotypes, we collected CNV genotypes for this locus in 450 samples from Conrad et al. [33], 1184 HapMap 3 samples [34] and 1092 from the most recent 1000 Genomes Phase 1 data release 20110521 [30]. The breakpoints of the CNV were characterized in a diverse set of individuals using primers by Sanger sequencing. The primers for PCR amplification can be found in Table S7. The overlapping CNV in HapMap 3 individuals is referred to as HM3_CNP_854 (hg18: chr22: 37,625,201–37,626,850). To ensure accuracy, we compared the genotypes of 411 shared samples between HapMap 3 [34] and Conrad et al. [33], and found very high concordance (99.5%). Overall, we were able to compile CNVR8163.1 deletion genotypes for a total of 1,723 individuals from 18 populations (Table S2).

### Selection analyses

To test for deviations from the neutral equilibrium model of evolution, Tajima's *D* [39] was calculated. Tajima's *D* is generally a measure of whether there are too few or too many rare variants at a given genomic locus. Significance values of *D* statistics were evaluated with 10,000 coalescent simulations using DNAsp version 5.10.01 [58]. We also applied *F*_ST_ statistics [59] to estimate population differentiation. Under an assumption of neutrality, *F*_ST_ is determined by demographic history and affects all loci similarly. Negative selection tends to decrease *F*_ST_, and positive selection tends to increase *F_ST_* [60]. At the NE1 locus, the *F_ST_* was calculated for each SNP. To evaluate the *F_ST_* level for the 36 kb LD block at the NE1 locus, we estimated *F_ST_* statistics between YRI and CEU for each non-overlapping 10 kb sliding window at the whole genome level.

The maximum likelihood HKA test was performed using multilocus data sets of 100 regions by the MLHKA software [61] using the number of segregating sites in the CEU population. Chimpanzee was used as an outgroup in this analysis. These 100 regions include the NE1 locus and ninety nine (99) 10 kb neutrally evolved regions, selected as described elsewhere [8]. The likelihood was evaluated under a neutral model and a selection model where the NE1 locus was subjected to natural selection. Statistical significance was assessed by a likelihood ratio test. We applied a chain length of 200,000 and repeated the program several times with different seeds to ensure stability of the results.

### Analysis of promoter activity of LTR regions

The full length LTR38-int fragment (2.3 kb) and the deleted LTR fragment (0.6 kb), from both NE1 and nonNE1 haplotypes, were PCR amplified using PFU Ultra II polymerase (Agilent Technologies) using DNA extracted from lymphoblastoid cell lines of individuals having homozygous NE1 and nonNE1 haplotypes. The fragments were confirmed by sequence analysis. Primers used for these experiments can be found in Table S7. To test for promoter function, the DNA fragments were cloned in front of the luciferase reporter sequence in the pGL3 basic vector (Promega). HEK 293T cells were transfected using polyethylenimine. Luciferase activity was measured 48h after transfection in cell lysates using a chemiluminesence assay (Promega). Experiments were performed in triplicates and replicated three times.

### ChIP–qPCR

Chromatin immunoprecipitation (ChIP) assays were performed as described previously [62]. Briefly, cells were cross-linked with 1% formaldehyde for 10 minutes. Chromatin lysates were then isolated and sonicated to generate fragments ranging from 300–600 bp. Immunoprecipitations were performed with 5 µg of anti-H3K4me2 (Millipore Cat#07-030) or an antibody recognizing choline acetyltransferase for a negative control. Antibody-chromatin complexes were isolated by Protein A beads. Immunoprecipitated chromatin was eluted with 1% SDS, cross-linking was reversed at 65°C, and then DNA was purified.

Purified DNA was quantitated by real-time PCR (qPCR) on a BioRad CFX96 Realtime System using a 5-point genomic DNA standard curve. The primers for these amplifications can be found in Table S7. qPCR buffer contained 5% dimethyl sulfoxide, 3 mM MgCl_2_, 20 mM Tris (pH 8.3), 50 mM KCl, 0.04% gelatin, 0.3% Tween-20, 1× SYBR green (Bio Whittaker Molecular Applications), 0.2 mM deoxynucleoside triphosphate, and 100 nM of each primer. All ChIP preparations were from four independent chromatin isolations, data averaged and plotted with respect to input chromatin.

### eQTL analyses

For the expression quantitative trait loci (eQTL) analyses, we utilized data from Illumina's commercial whole genome expression array, Sentrix Human-6 Expression BeadChip version 2. These arrays utilize a bead pool with ∼48,000 unique bead types (one for each of 47,294 transcripts, plus controls), each with several hundred thousand gene-specific 50mer probes attached. Of the 47,294 probes where expression data were available, we selected a set of 21,800 probes to analyze. We included in our analyses each probe that mapped to an Ensembl gene, but not to more than one Ensembl gene (Ensembl 49 NCBI Build 36) for probes in autosomal chromosomes. We excluded probes mapping to the X or Y chromosome as splitting the sample set to male and female cohorts would significantly reduce the power of our analysis. The resulting set of 21,800 probes was subjected to association analyses, corresponding to 18,226 unique autosomal Ensembl genes. We tested these associations with all of the SNP genotypes regardless of the haplogroup in 109 CEU, 162 CHB/JPT and 108 YRI samples located within the 36 kb region. Using Spearman Rank Correlation (SRC) to associate allele count (coded as 0,1,2) with normalized gene expression levels, we performed ∼3.5 million tests per population. None of the *trans*-eQTL associations were significant using a strict Bonferroni multiple hypothesis testing correction. To test for any *cis*-eQTL associations, we used SRC for associations between genotypes of every SNP that fell into our haplotype block and expression levels of any gene where that gene's transcription start site was less than 1 Mb up- or downstream of the SNP. We provide the p-values for these *cis* associations in the CEU and CHB/JPT populations in Table S8.

## Supporting Information

Figure S1 LD patterns in different populations at the NE1 locus. Populations shown include: CEU (European ancestry from UTAH), FIN (Finnish ancestry), GBR (Briton ancestry), TSI (Tuscan ancestry), CHB (Chinese ancestry from Beijing), CHS (Han Chinese South), JPT (Japanese ancestry from Tokyo), YRI (Yoruban ancestry from Ibadan), ASW (African ancestry from Southwestern US), LWK (Luhya ancestry from Kenya).
(TIF)

Figure S2 Nucleotide diversity and segregating sites at the NE1 locus among 180 CEU haplotypes as compared with other similarly sized loci across the genome. The left panel shows the nucleotide diversity (π), estimated for each 10 kb sliding window among 180 CEU haplotypes. The y-axis represents the frequency of segments with a given π value. The vertical dotted line indicates the π value at the NE1 locus. The probability of observing a π value similar or greater than that found for the NE1 locus is significantly low (p = 0.00049). The right panel depicts the number of segregating sites that was estimated for each 10 kb sliding window across the genomes of 90 CEU individuals. The y-axis represents the frequency of observations with a given number of segregating sites. The vertical dotted line indicates the number of segregating sites observed at the NE1 locus. The number of segregating sites in NE1 locus is significantly higher than expected by chance alone (p = 0.00142). The inlaid barplot on the right indicates that there are more SNPs (“segregating sites”) among nonNE1 haplotypes as compared to NE1 haplotypes.
(TIF)

Figure S3 Pairwise differences between European and African populations as compared to Neandertal haplotypes. The pairwise differences between Neandertal haplotype and CEU population are minimal in comparison to differences between Neandertal haplotype and YRI. We assessed a total of 209 segregating sites obtained from the Neandertal reference genome sequence that aligns with the human NE1 locus. For the leftmost box, we calculated the pairwise distances of each haplotype in the CEU population to those in the YRI population. For the other two boxes, we calculated the pairwise distance to the Neandertal haplotype as deduced from the Neandertal reference genome alignment in the UCSC Genome browser. p-values were calculated using Mann-Whitney test.
(TIF)

Figure S4 Frequency of NE1 and nonNE1 haplogroups in the Human Genome Diversity Panel populations. The panel summarizes the frequency of NE1 and nonNE1 haplogroups in each of the 33 populations. *NE1* and *nonNE1* haplogroups are denoted on the X-axis. Frequency (in percent) is denoted on the Y-axis. Specific haplotypes are color coded as depicted on the far right. The haplotypes were curated for the SNPs rs11913682, rs4361209, rs132500, rs2142836, rs469987, rs2413552, respectively. The phased haplotypes were downloaded from http://www.stanford.edu/group/rosenberglab/diversity.html#data4. Of note, we successfully assigned all the common haplotypes to the two haplogroups with the exception of two singleton haplotypes, AAGGTA and GAGGCA, which were omitted from this analysis.
(TIF)

Figure S5 PCA of NE1 and nonNE1 haplotypes in worldwide populations. The nonNE1 (left) and NE1 (right) haplotypes separate across PC1, regardless of the population of origin. Please note the wide separation of African haplotypes (blue) across PC2, both within NE1 and within nonNE1 haplogroups.
(TIF)

Figure S6 Network Analysis. This network shows clear separation of the NE1 and nonNE1 haplotypes with multiple mutations, a hallmark of balancing selection. We have calculated the Median Joining Network of the phased haplotypes for the NE1 locus. The left panel shows the nodes represented by common haplotypes (>2 haplotypes). On the right panel, the haplotypes that belong to NE1 node are depicted. The African haplotypes are shown by shades of blue as shown in the label key. The bar plot shows the age estimations of NE1 coalescence based on this network. Specifically, we assumed a generation time of 20 and used two different mutation rates (2.5×10^−8^ and 1.1×10^−8^ mutations per site per year) and found coalescence dates of ∼993 K years before present (YBP) and ∼437 K YBP for African NE1 haplotypes, respectively. The European NE1 coalescence is much more recent at 304 K YBP and ∼134 K YBP. For the coalescence date for NE1 and nonNE1, we estimated 4,639 K YBP and 2,041 K YBP. These results are concordant with our estimations in Figure 4A and are in keeping with the idea that NE1 haplogroup in modern humans predates introgression from ancient hominins.
(TIF)

Figure S7 Comparison of the Tajima's *D* statistics observed at the NE1 locus with the distribution of Tajima's *D* values across the human genome for the CEU and YRI populations. Tajima's *D* is estimated for each 10 kb window. The y-axis represents the frequency for a given Tajima's *D* value. The red vertical line indicates the Tajima's *D* values at the NE1 locus for each of these populations. NE1 locus show a significantly larger Tajima's D value for the CEU population, but not for the YRI. Empirical p-values are shown for each population adjacent to the dotted line.
(TIF)

Figure S8 Comparison of the *F_ST_* values between CEU and YRI for the NE1 locus. The distribution of *F_ST_* values across the human genome is calculated between these two populations using 10 kb bins. The y-axis represents the density of segments with a given *F_ST_* value. The red vertical line indicate the *F_ST_* values at the NE1 locus, which is significantly higher than genome-wide distribution (p = 0.00285).
(TIF)

Figure S9 Promoter activity of the NE1 locus measured by luciferase reporter assay. “Full length LTR”, “Deleted LTR nonNE1” and “Deleted LTR NE1” indicate the portion of the region and the haplotype cloned into pGL3 reporter assays. The nonNE1 and NE1 haplotypes have 2 SNPs changing the sequences of “Deleted LTR nonNE1” (Blue) and “Deleted LTR NE1” (orange). Please note that the former sequence, which has the observed promoter activity, exists only in the presence of the remainder of the LTR fragment in human populations and, as a whole, do not show promoter activity. These regions were cloned into pGL3 basic luciferase reporter vector and luminescence was measured in Relative Luminescence Units (RLU) 48 h after transfection into HEK293T cells (data plotted is representative of two experiments in triplicate, +/− SD). The full “LTR38-int” from nonNE1 haplotypes (“Full length LTR”) does not have a promoter activity. We noticed that the 622 nucleotide LTR38-int fragment outside the deletion boundaries harbors six SNPs that are fixed differences between NE1 and nonNE1 haplotypes which may aid in suppressing the regulatory activity of the “Deleted LTR NE1” sequence (p<0.01).
(TIF)

Figure S10 The −log distribution of p-values for the genes associated with variation at the NE1 locus for CEU, CHB/JPT and YRI populations. The p-values were calculated using Spearman Rank Correlation (SRC) and subsequent permutation testing. The strongest SNP-Gene associations are indicated with the blue vertical lines. The gene associations with SNPs that segregate perfectly between NE1 and nonNE1 haplogroups are indicated with the vertical red lines. There are no eQTLs that are consistent significant between the different populations. Of note, the YRI population has very few deletion haplotypes and we would likely lack sufficient power to detect eQTL associations in this population, even if such associations exist. The strong associations presented here for YRI are all for variants seen within nonNE1 haplotypes.
(TIF)

Table S1 12 SNPs tagging deletion CNVR8136.1. Genomic positions are based on hg18 (Build 36, March 2006) coordinates.
(XLSX)

Table S2 Worldwide distribution of CNVR8136.1 genotype. Genotypes in the Column C were downloaded from Hapmap 3 (http://hapmap.ncbi.nlm.nih.gov/). Genotypes in the Column D were extracted from Conrad et al (2010). Genotypes in the column E were speculated based on 12 fixed SNPs listed in Table S1.
(XLSX)

Table S3 Tajima's *D* statistics. * P< = 0.05. ** P< = 0.01.
(XLSX)

Table S4 *F_ST_* values among populations for NE1 locus. SNPs of 12 populations were collected from 1000 Genomes phase1. 3 dataset (all haplotypes, haplotypes containing CNVR8163.1 deletions and haplotypes without CNVR8163.1 deletions) were generated for each population. For instance, ASW represents all haplotypes from this population; ASW_NE1 represents ASW haplotypes with CNVR8163.1 deletions and ASW_NonNE1 represents haplotypes without CNVR8163.1 deletions. All YRI haplotypes don't contain CNVR8163.1 deletion, and one haplotype from the PUR population containing the CNVR8163.1 deletion was excluded from this analysis.
(XLSX)

Table S5 The number of segregating sites calculated for the HKA test.
(XLSX)

Table S6 Hg18 coordinates of regions that show similar nucleotide diversity, population differentiation and site frequency spectrum. Two regions in the first 2 rows from chromosome 11 are adjacent to each other, denoting a single, larger locus with those properties.
(XLSX)

Table S7 Primers used in this study.
(XLSX)

Table S8 *cis* association for CEU and CHBJPT populations.
(XLSX)
