# Supplementary figures and images for: Balancing Selection on a Regulatory Region Exhibiting Ancient Variation That Predates Human–Neandertal Divergence
Source: PLoS Genet. 2013 Apr 11;9(4):e1003404. doi: 10.1371/journal.pgen.1003404 (PMC3623772; doi:10.1371/journal.pgen.1003404)

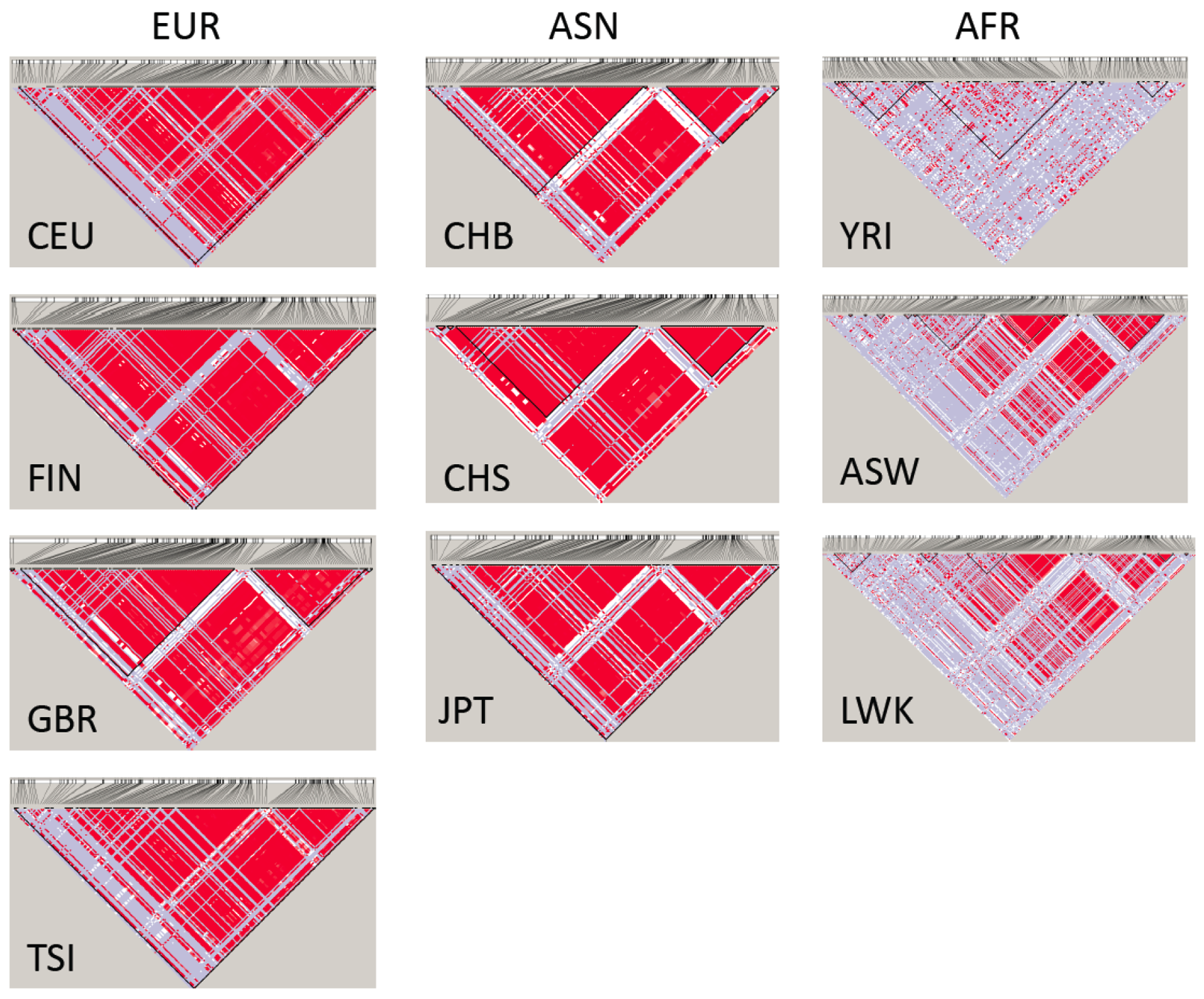

Supplement: Figure S1 — LD patterns in different populations at the NE1 locus. Populations shown include: CEU (European ancestry from UTAH), FIN (Finnish ancestry), GBR (Briton ancestry), TSI (Tuscan ancestry), CHB (Chinese ancestry from Beijing), CHS (Han Chinese South), JPT (Japanese ancestry from Tokyo), YRI (Yoruban ancestry from Ibadan), ASW (African ancestry from Southwestern US), LWK (Luhya ancestry from Kenya). (TIF) [file pgen.1003404.s001.tif]

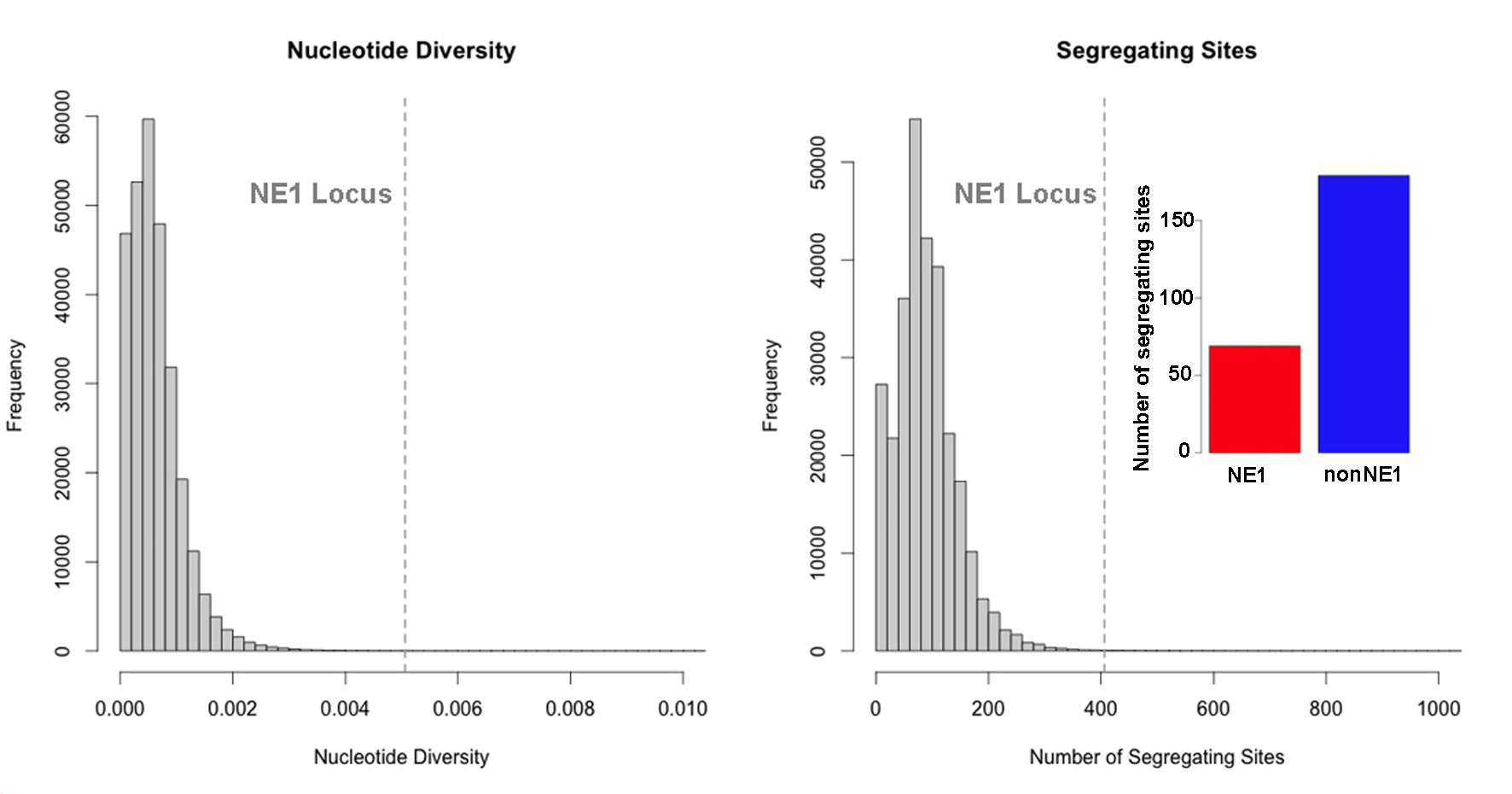

Supplement: Figure S2 — Nucleotide diversity and segregating sites at the NE1 locus among 180 CEU haplotypes as compared with other similarly sized loci across the genome. The left panel shows the nucleotide diversity (π), estimated for each 10 kb sliding window among 180 CEU haplotypes. The y-axis represents the frequency of segments with a given π value. The vertical dotted line indicates the π value at the NE1 locus. The probability of observing a π value similar or greater than that found for the NE1 locus is significantly low (p = 0.00049). The right panel depicts the number of segregating sites that was estimated for each 10 kb sliding window across the genomes of 90 CEU individuals. The y-axis represents the frequency of observations with a given number of segregating sites. The vertical dotted line indicates the number of segregating sites observed at the NE1 locus. The number of segregating sites in NE1 locus is significantly higher than expected by chance alone (p = 0.00142). The inlaid barplot on the right indicates that there are more SNPs (“segregating sites”) among nonNE1 haplotypes as compared to NE1 haplotypes. (TIF) [file pgen.1003404.s002.tif]

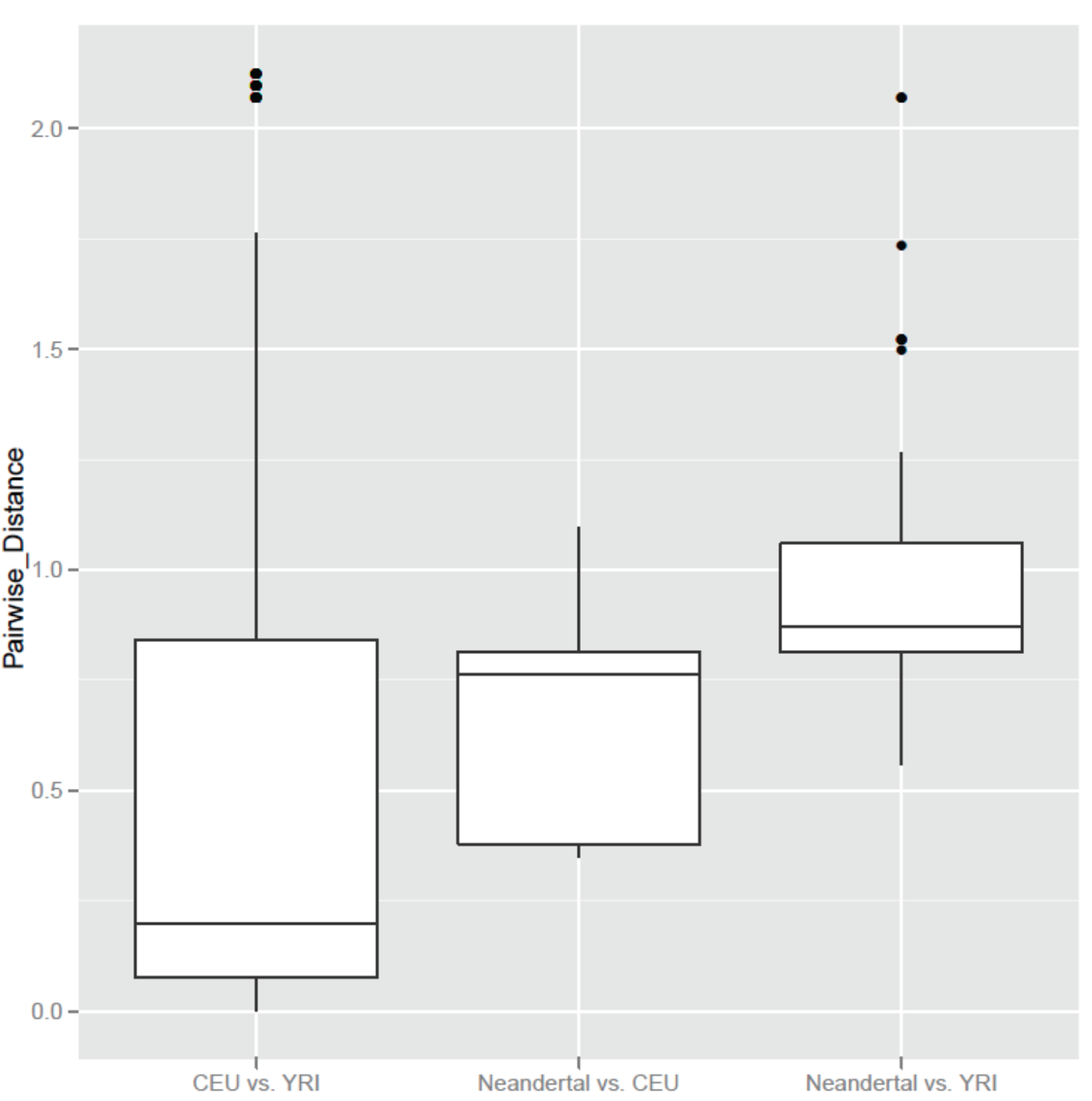

Supplement: Figure S3 — Pairwise differences between European and African populations as compared to Neandertal haplotypes. The pairwise differences between Neandertal haplotype and CEU population are minimal in comparison to differences between Neandertal haplotype and YRI. We assessed a total of 209 segregating sites obtained from the Neandertal reference genome sequence that aligns with the human NE1 locus. For the leftmost box, we calculated the pairwise distances of each haplotype in the CEU population to those in the YRI population. For the other two boxes, we calculated the pairwise distance to the Neandertal haplotype as deduced from the Neandertal reference genome alignment in the UCSC Genome browser. p-values were calculated using Mann-Whitney test. (TIF) [file pgen.1003404.s003.tif]

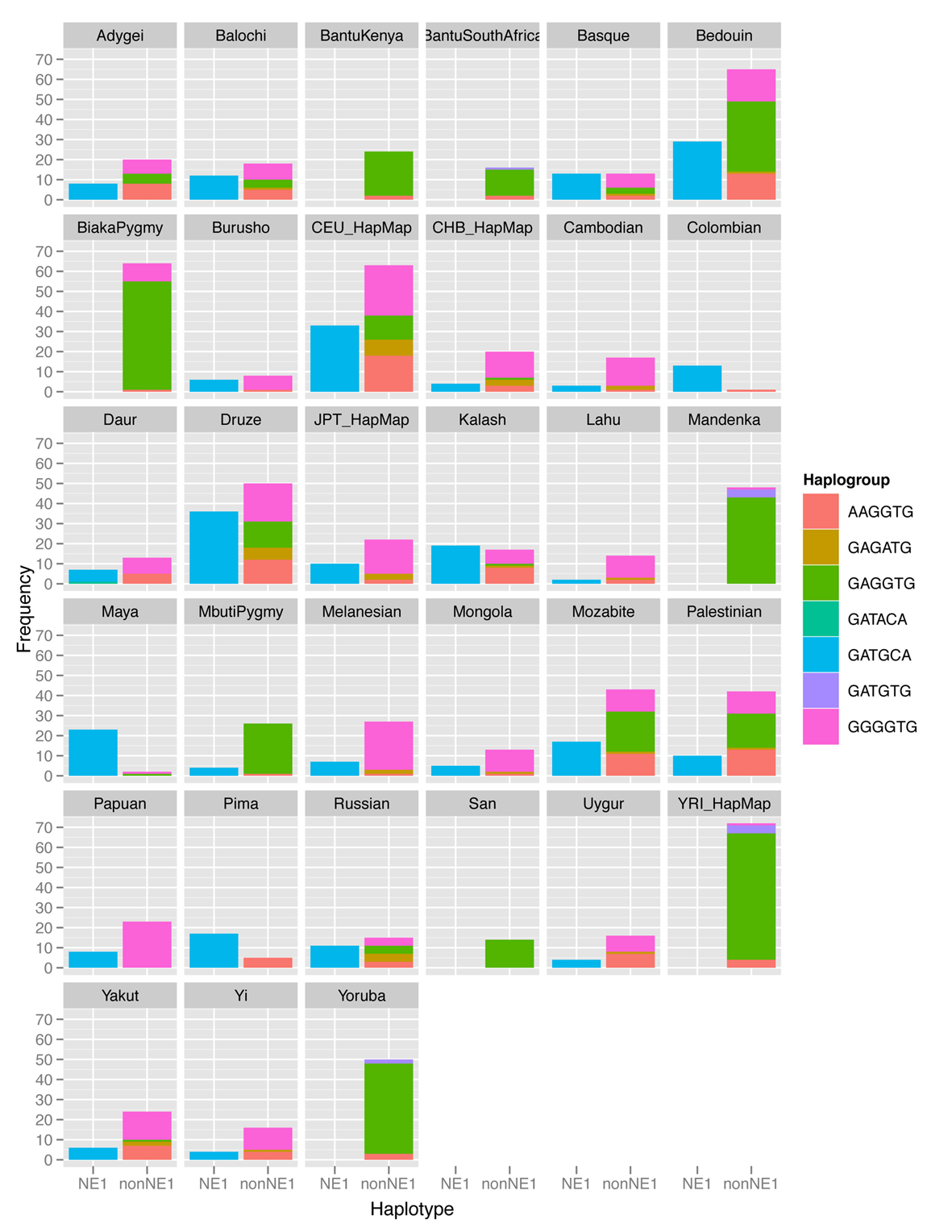

Supplement: Figure S4 — Frequency of NE1 and nonNE1 haplogroups in the Human Genome Diversity Panel populations. The panel summarizes the frequency of NE1 and nonNE1 haplogroups in each of the 33 populations. NE1 and nonNE1 haplogroups are denoted on the X-axis. Frequency (in percent) is denoted on the Y-axis. Specific haplotypes are color coded as depicted on the far right. The haplotypes were curated for the SNPs rs11913682, rs4361209, rs132500, rs2142836, rs469987, rs2413552, respectively. The phased haplotypes were downloaded from http://www.stanford.edu/group/rosenberglab/diversity.html#data4. Of note, we successfully assigned all the common haplotypes to the two haplogroups with the exception of two singleton haplotypes, AAGGTA and GAGGCA, which were omitted from this analysis. (TIF) [file pgen.1003404.s004.tif]

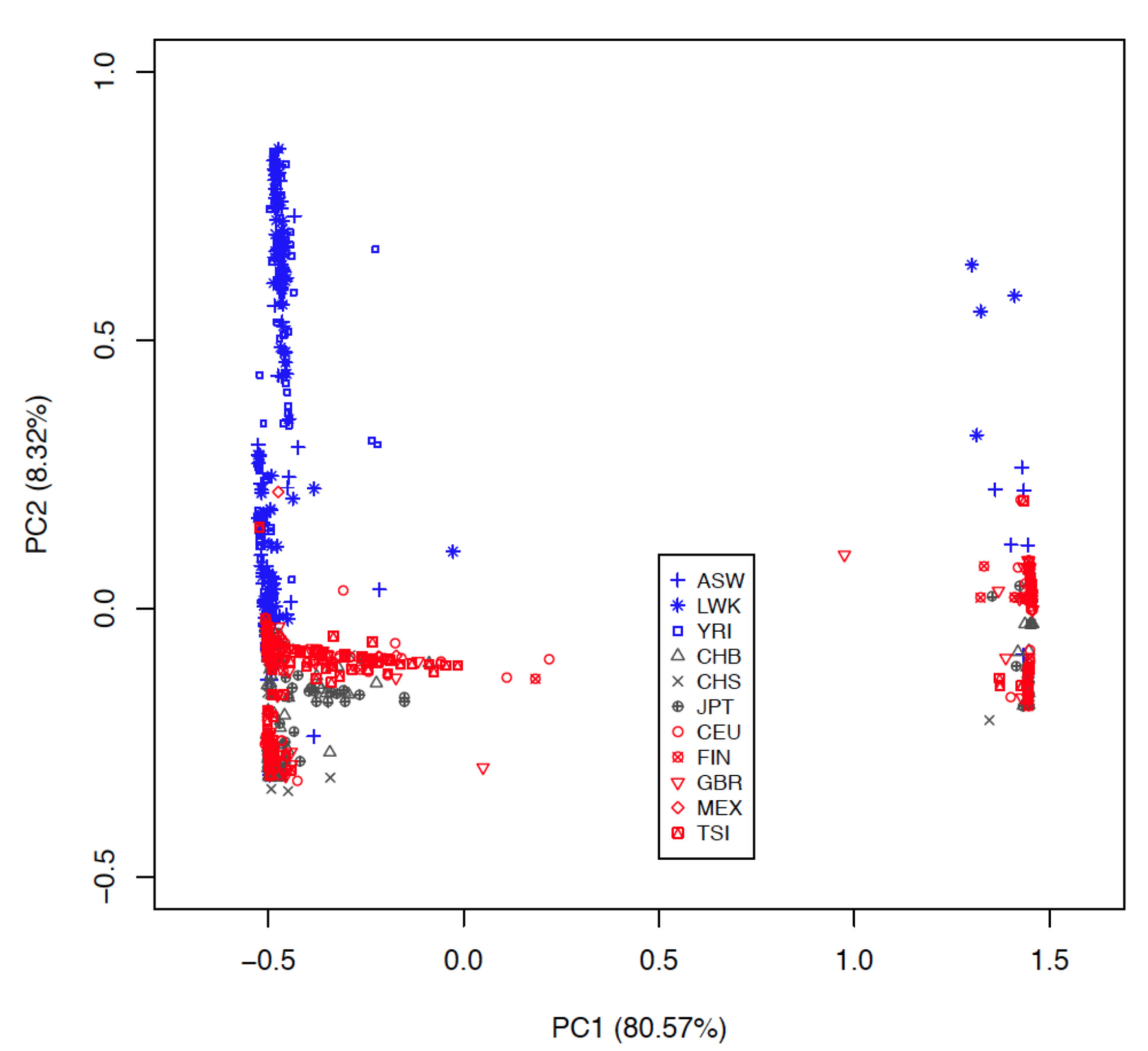

Supplement: Figure S5 — PCA of NE1 and nonNE1 haplotypes in worldwide populations. The nonNE1 (left) and NE1 (right) haplotypes separate across PC1, regardless of the population of origin. Please note the wide separation of African haplotypes (blue) across PC2, both within NE1 and within nonNE1 haplogroups. (TIF) [file pgen.1003404.s005.tif]

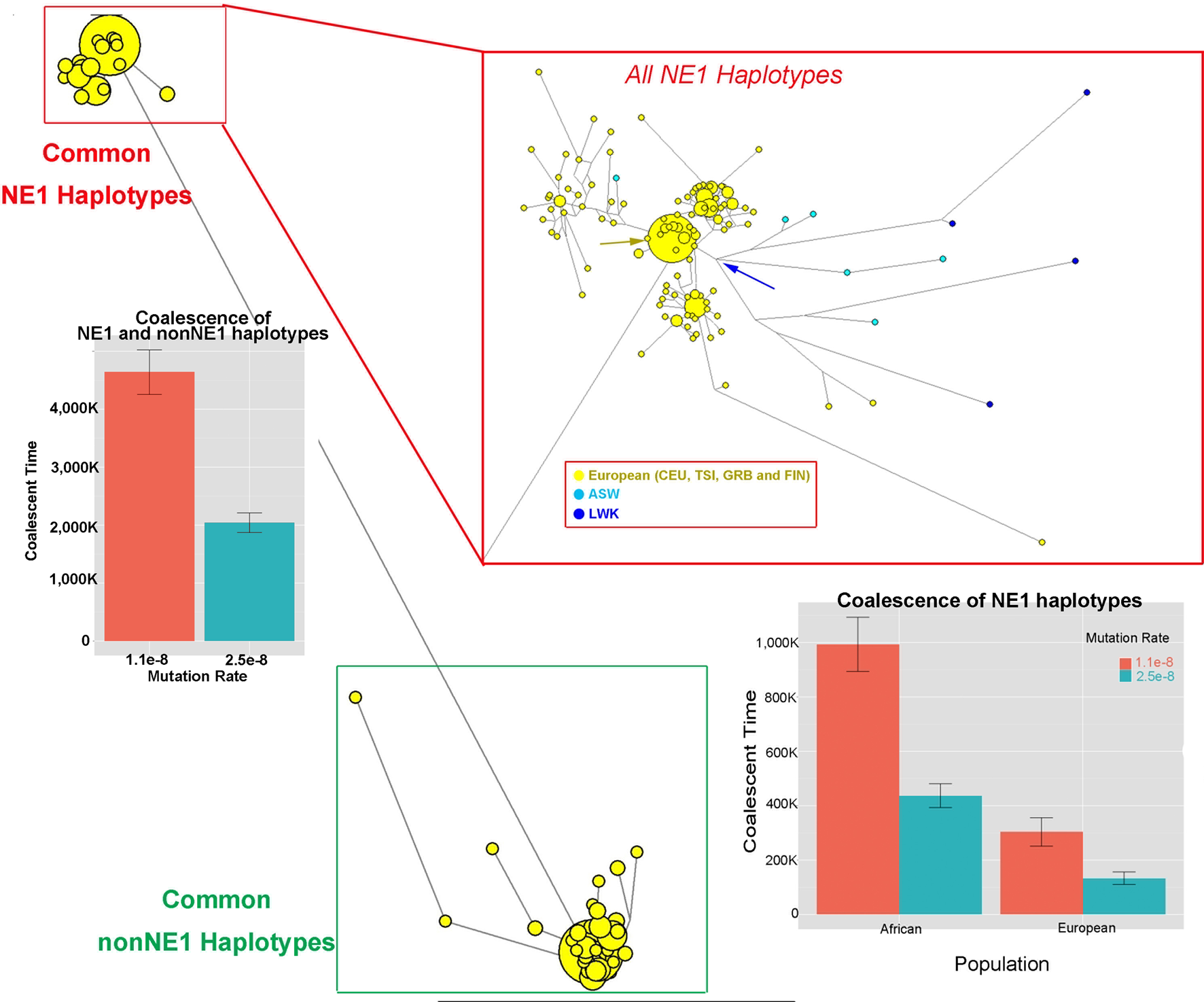

Supplement: Figure S6 — Network Analysis. This network shows clear separation of the NE1 and nonNE1 haplotypes with multiple mutations, a hallmark of balancing selection. We have calculated the Median Joining Network of the phased haplotypes for the NE1 locus. The left panel shows the nodes represented by common haplotypes (>2 haplotypes). On the right panel, the haplotypes that belong to NE1 node are depicted. The African haplotypes are shown by shades of blue as shown in the label key. The bar plot shows the age estimations of NE1 coalescence based on this network. Specifically, we assumed a generation time of 20 and used two different mutation rates (2.5×10^−8^ and 1.1×10^−8^ mutations per site per year) and found coalescence dates of ∼993 K years before present (YBP) and ∼437 K YBP for African NE1 haplotypes, respectively. The European NE1 coalescence is much more recent at 304 K YBP and ∼134 K YBP. For the coalescence date for NE1 and nonNE1, we estimated 4,639 K YBP and 2,041 K YBP. These results are concordant with our estimations in Figure 4A and are in keeping with the idea that NE1 haplogroup in modern humans predates introgression from ancient hominins. (TIF) [file pgen.1003404.s006.tif]

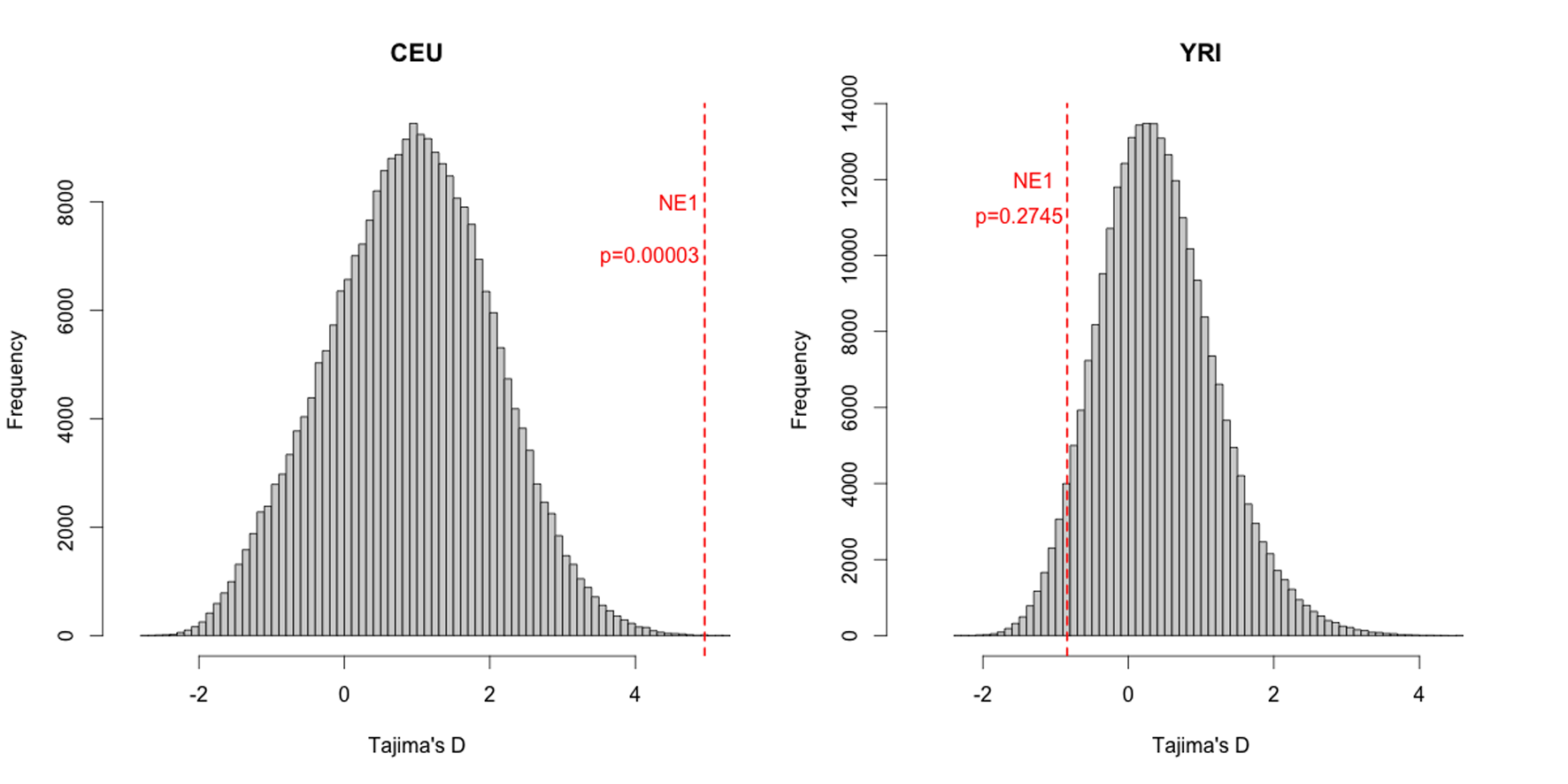

Supplement: Figure S7 — Comparison of the Tajima's D statistics observed at the NE1 locus with the distribution of Tajima's D values across the human genome for the CEU and YRI populations. Tajima's D is estimated for each 10 kb window. The y-axis represents the frequency for a given Tajima's D value. The red vertical line indicates the Tajima's D values at the NE1 locus for each of these populations. NE1 locus show a significantly larger Tajima's D value for the CEU population, but not for the YRI. Empirical p-values are shown for each population adjacent to the dotted line. (TIF) [file pgen.1003404.s007.tif]

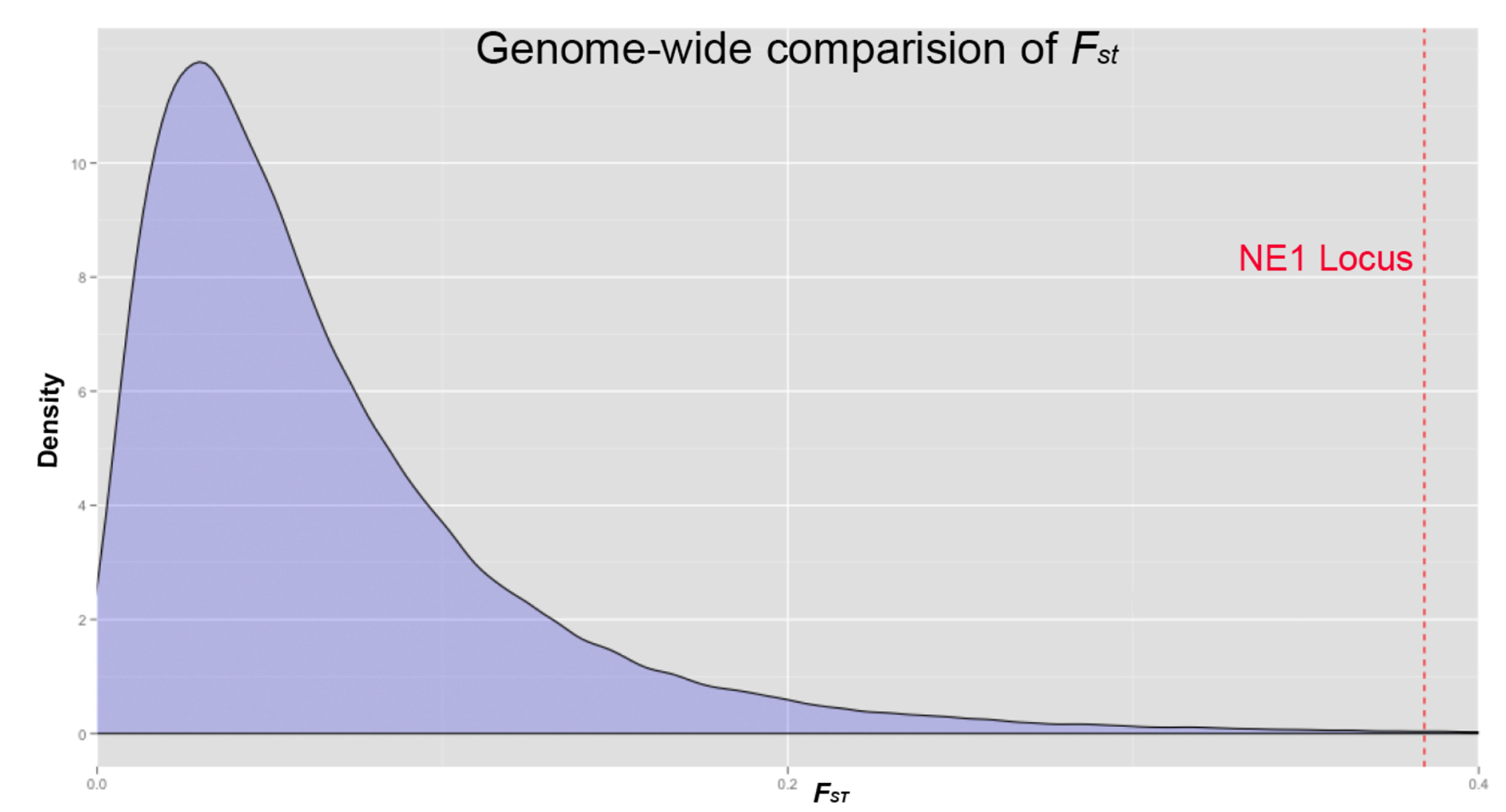

Supplement: Figure S8 — Comparison of the FST values between CEU and YRI for the NE1 locus. The distribution of FST values across the human genome is calculated between these two populations using 10 kb bins. The y-axis represents the density of segments with a given FST value. The red vertical line indicate the FST values at the NE1 locus, which is significantly higher than genome-wide distribution (p = 0.00285). (TIF) [file pgen.1003404.s008.tif]

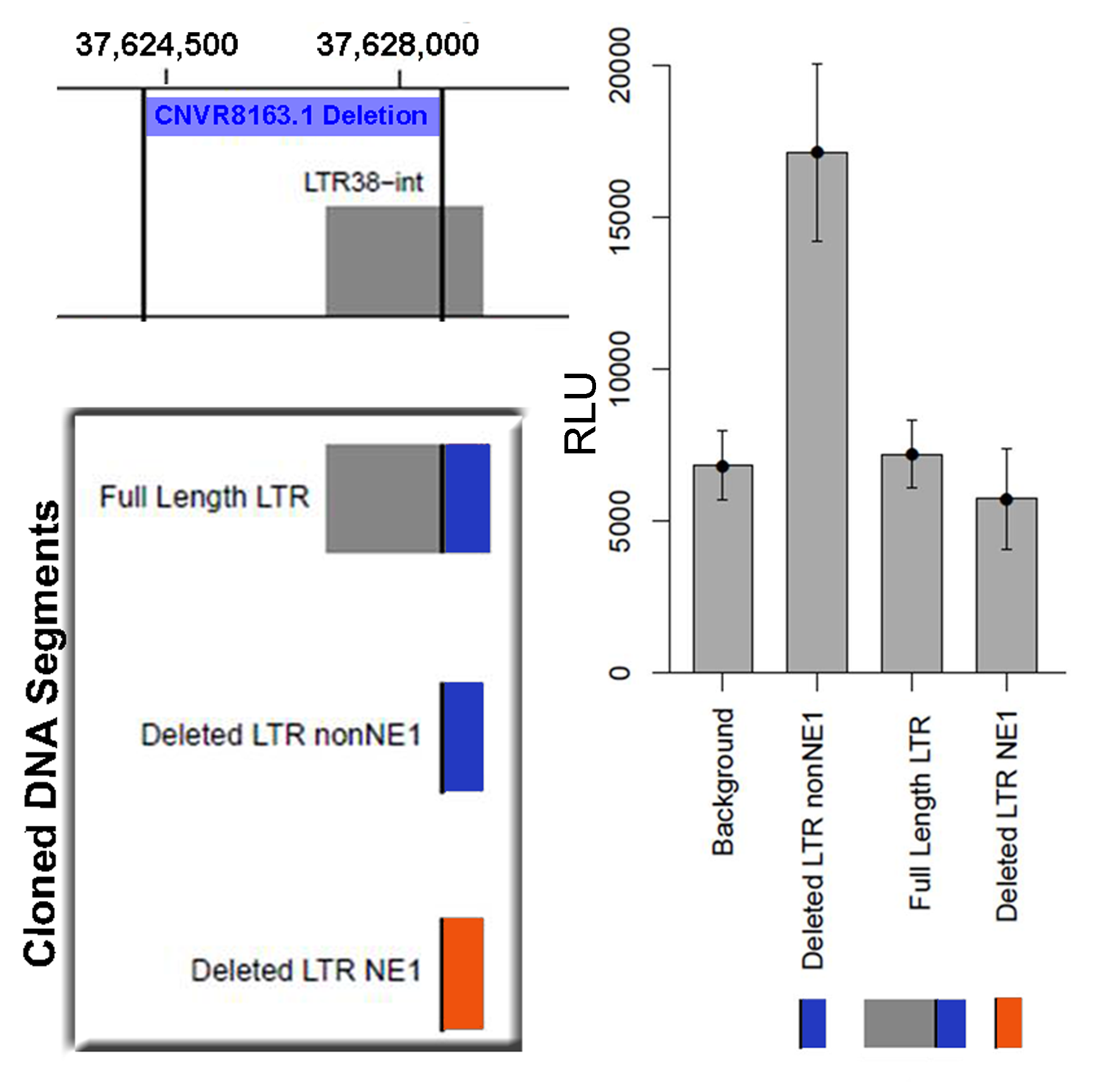

Supplement: Figure S9 — Promoter activity of the NE1 locus measured by luciferase reporter assay. “Full length LTR”, “Deleted LTR nonNE1” and “Deleted LTR NE1” indicate the portion of the region and the haplotype cloned into pGL3 reporter assays. The nonNE1 and NE1 haplotypes have 2 SNPs changing the sequences of “Deleted LTR nonNE1” (Blue) and “Deleted LTR NE1” (orange). Please note that the former sequence, which has the observed promoter activity, exists only in the presence of the remainder of the LTR fragment in human populations and, as a whole, do not show promoter activity. These regions were cloned into pGL3 basic luciferase reporter vector and luminescence was measured in Relative Luminescence Units (RLU) 48 h after transfection into HEK293T cells (data plotted is representative of two experiments in triplicate, +/− SD). The full “LTR38-int” from nonNE1 haplotypes (“Full length LTR”) does not have a promoter activity. We noticed that the 622 nucleotide LTR38-int fragment outside the deletion boundaries harbors six SNPs that are fixed differences between NE1 and nonNE1 haplotypes which may aid in suppressing the regulatory activity of the “Deleted LTR NE1” sequence (p<0.01). (TIF) [file pgen.1003404.s009.tif]

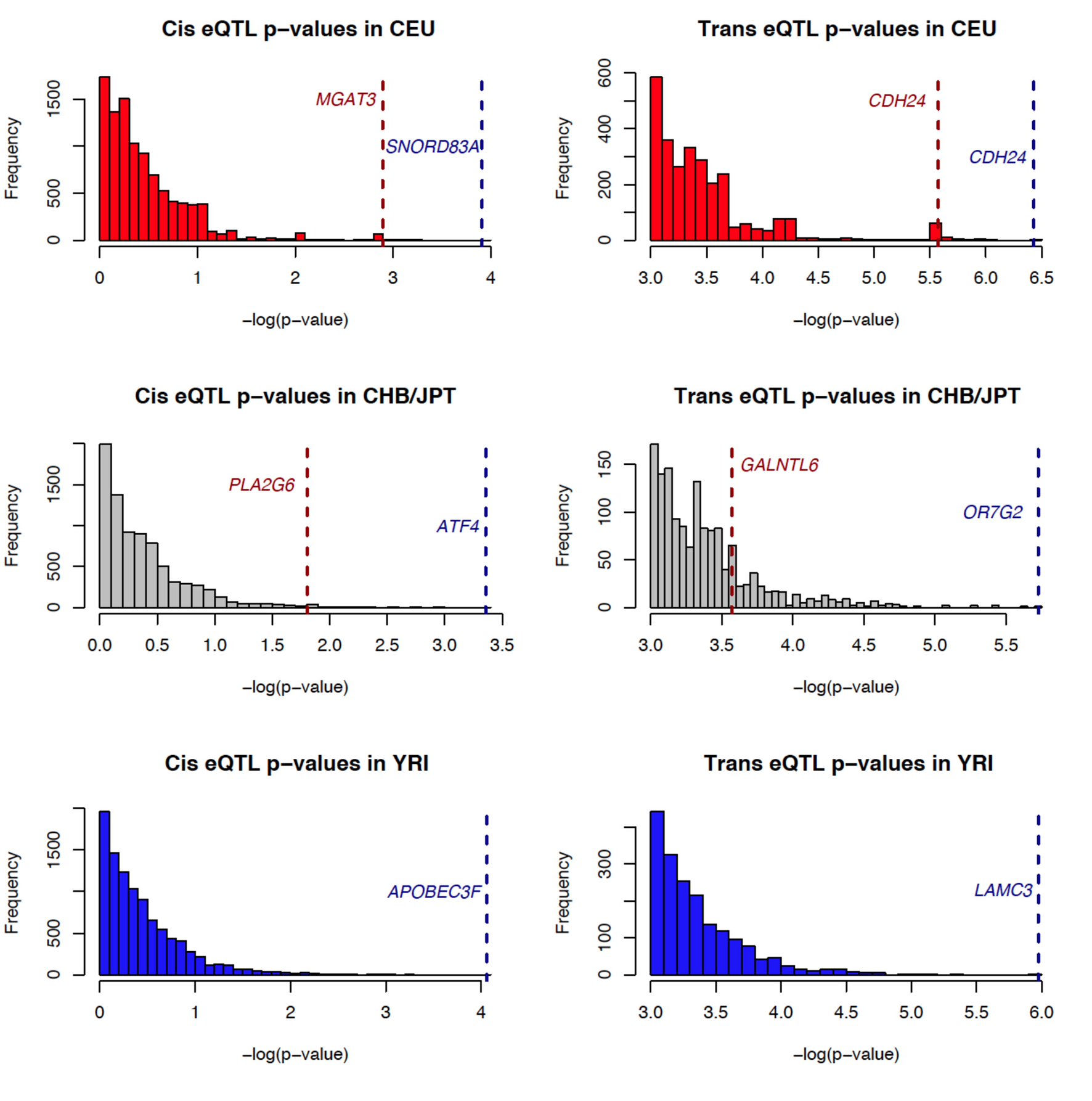

Supplement: Figure S10 — The −log distribution of p-values for the genes associated with variation at the NE1 locus for CEU, CHB/JPT and YRI populations. The p-values were calculated using Spearman Rank Correlation (SRC) and subsequent permutation testing. The strongest SNP-Gene associations are indicated with the blue vertical lines. The gene associations with SNPs that segregate perfectly between NE1 and nonNE1 haplogroups are indicated with the vertical red lines. There are no eQTLs that are consistent significant between the different populations. Of note, the YRI population has very few deletion haplotypes and we would likely lack sufficient power to detect eQTL associations in this population, even if such associations exist. The strong associations presented here for YRI are all for variants seen within nonNE1 haplotypes. (TIF) [file pgen.1003404.s010.tif]
